# Diffuse persistent pulmonary interstitial emphysema secondary to mechanical ventilation in bronchiolitis

**DOI:** 10.1186/s12890-016-0299-9

**Published:** 2016-11-03

**Authors:** Blanca Toledo del Castillo, Isabel Gordillo, Elena Rubio García, Sarah Nicole Fernández Lafever, Rafael Gonzalez Cortés, Javier Urbano Villaescusa, Jorge López González, María José Solana García, Jesús López-Herce Cid

**Affiliations:** 1 Pediatric Intensive Care Department, Hospital General Universitario Gregorio Marañón, Red de Investigación en Salud Materno-Infantil y del Desarrollo (Red SAMYD), C/ Doctor Castelo 47, 28009 Madrid, Spain; 2 Radiology Department, Hospital General Universitario Gregorio Marañón, C/ Doctor Castelo 47, 28009 Madrid, Spain; 3 Pediatric Department, Hospital General Universitario Gregorio Marañón, C/ Doctor Castelo 47, 28009 Madrid, Spain

**Keywords:** Diffuse persistent interstitial emphysema, Mechanical ventilation, Neonatal, ECMO

## Abstract

**Background:**

Persistent interstitial pulmonary emphysema (PIE) is a rare disease and it is even more uncommon in full-term infants, like our patient. When conservative management is not successful, surgical treatment should be considered. In our case, ECMO support was iniciated to keep the patient ventilated in order to allow the lung to heal using lung protection strategies.

**Case presentation:**

We report an 18-day-old male infant with bronchiolitis that required mechanical ventilation with high positive airway pressures due to severe respiratory insufficiency. Chest X-rays and computed tomography scan revealed a severely hyperinflated left lung with extensive destructive changes and multiple small bullae. These findings were consistent with diffuse persistent interstitial emphysema (PIE), probably due to mechanical ventilation. The patient required high frequency oscillatory ventilation, inotropic support and continuous renal replacement therapy. He eventually suffered a cardiac arrest that required cardiopulmonary resuscitation and ECMO during 5 days with progressive clinical improvement and normalization of the X-ray.

**Conclusion:**

We present a patient with diffuse persistent interstitial emphysema who, despite an unfavorable evolution with different mechanical ventilation strategies, had a good response after ECMO assistance.

## Background

Persistent interstitial pulmonary emphysema (PIE) is a rare disease. Very few cases have been reported in premature newborns without invasive mechanical ventilation, and it is even more uncommon in full-term infants, like our patient. When conservative management is not successful, surgical treatment should be considered. In our case, due to clinical instability after a cardiac arrest, we decided to initiate ECMO support and to keep the patient ventilated in order to allow the lung to heal using lung protection strategies. There are no reports on the use of ECMO in patients with PIE even though its efficacy was described a long time ago.

## Case presentation

**Fig. 1:**
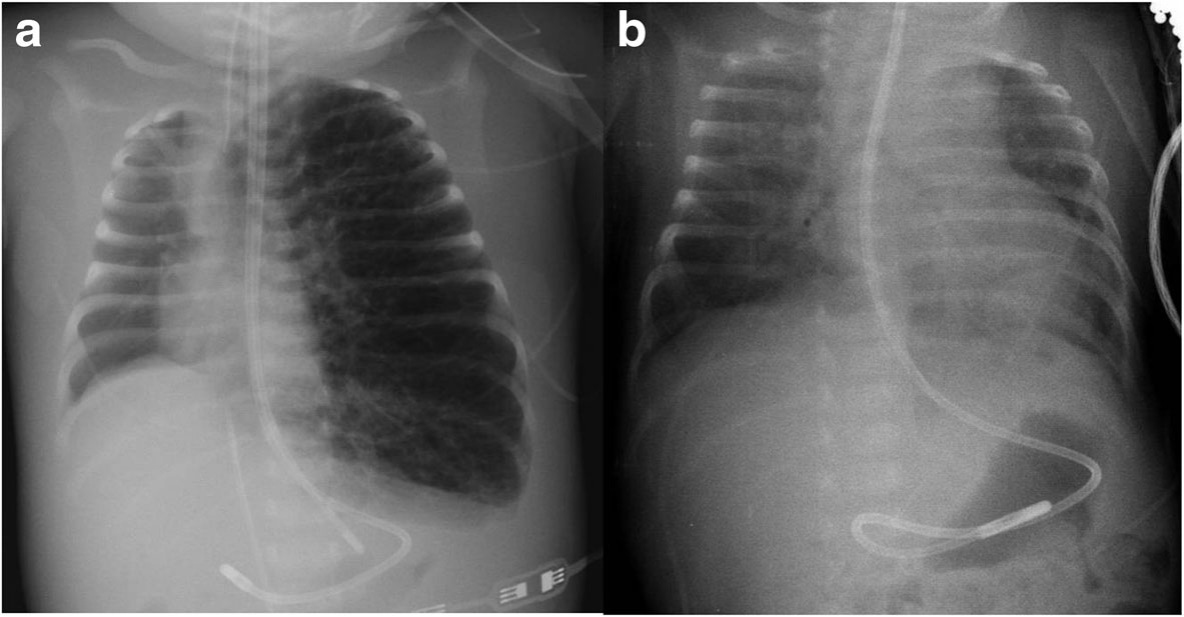
Chest X-Rays showing left lung hyperinflation with cystic images on the day of intubation **a** and a marked improvement after 5 days of ECMO support **b**

An 18-day-old male infant with no risk factors was admitted to our hospital due to bronchiolitis caused by Respiratory Sincitial Virus. The initial X-ray showed upper right lobe atelectasis. He received high flow oxygen therapy for 5 days (maximum 10 lpm and FiO2 80 %), but he developed progressive respiratory insufficiency, without signs of bacterial infection. He was admitted to the PICU and required intubation and mechanical ventilation. Chest X-ray at this moment showed unilateral hyperinflation with cystic images (Fig. 1a). He presented progressive hypoventilation with severe hypercapnia (maximum PaCO_2_ 102 mmHg) and air trapping with autoPEEP of 10 mmHg. He required mechanical ventilation with high pressures (40 mmHg), despite several pulmonary protective strategies: mechanical ventilation with low volumes and long expiratory time, high frequency oscillatory ventilation (HFOV) with maximum settings (Mean airway pressure 15 mmHg, Frequency 8 Hz, Amplitude 45 cmH2O), heliox administration, inhaled nitric oxide, lateral position and selective tracheal intubation.

**Fig. 2:**
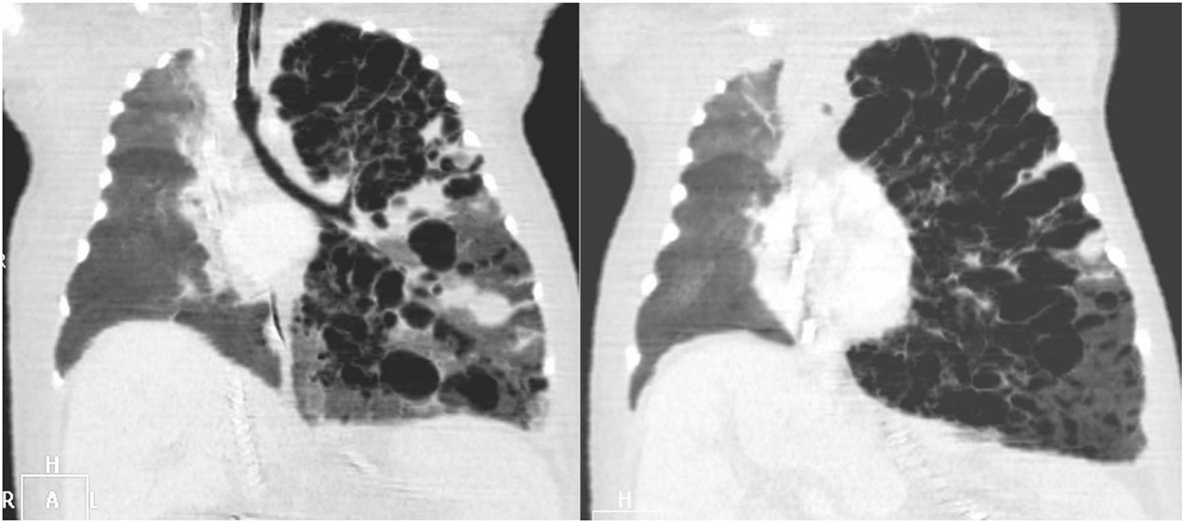
Severely hyperinflated left lung with extensive destructive changes and multiple small bullae

The differential diagnosis was made with other cystic lesions such as congenital cystic malformations, cystic lymphangioma and congenital lobar emphysema. Chest computed tomographic scan (CT scan) revealed a severely hyperinflated left lung with extensive destructive changes and multiple small bullae. The left lung was markedly herniated to the right with partial collapse of the right lung and marked deviation of the mediastinum and heart to the right (Fig. 2). These findings were consistent with diffuse interstitial emphysema (PIE). The patient didn’t show clinical or analytical signs of infection and blood and respiratory cultures were negative. For this reason, the possibility of a necrotizing pneumonia due to a bacterial infection was ruled out. Echocardiographic study was normal.

He required inotropic support (dopamine, milrinone, adrenaline and noradrenaline) and continuous renal replacement therapy due to hemodynamic instability and acute kidney injury. On the 17^th^ day after admission he suffered a cardiac arrest that required cardiopulmonary resuscitation. After return of spontaneous circulation, we decided to start veno-arterial Extracorporeal Membrane Oxigenation (ECMO) support, through cervical cannulation. It was maintained during 5 days while he was ventilated with very low pressures. The patient showed progressive improvement in ventilation and oxygenation parameters and X-ray images (Fig. 1b). He was extubated 2 days after the removal of ECMO assistance, after 23 days of mechanical ventilation. One month later, the patient was finally discharged without any supplemental oxygen and the chest X-ray returned to normal. He presented a good neurologic and respiratory evolution. One year later, the neurologic exploration and cerebral magnetic nuclear resonance were normal.

Persistent interstitial pulmonary emphysema (PIE) is a rare disease that is usually seen in preterm infants with mechanical ventilation. The perivascular connective tissue in preterm infants is more abundant and less dissective and air can be trapped in the perivascular space. Other risk factors are surfactant deficiency syndrome, meconium aspiration syndrome and infection [1].

There are only a few reported cases of PIE developing in premature newborns without invasive mechanical ventilation [1]. Although it is not common, it has also been reported in full-term infants. In our patient, PIE initiated during HFO therapy but got worse with mechanical ventilation, probably due to the high pressures that were needed to ventilate the patient.

PIE has been described as acute or persistent and local or diffuse. Our patient presented diffuse emphysema because there were smaller cyst in all the lobes of the left lung, and it was persistent in the time. The mechanism of production of PIE is the disruption of the alveolar wall basement membrane with subsequent dissection of air into the interstitial space [2].

The definitive diagnosis typically requires chest computed tomography. Differential diagnosis must be made with some congenital and acquired diseases as congenital pulmonary airway malformation, bronchogenic cyst, congenital lobar emphysema, lympahngiectasia, cystic lymphangioma and sequelae to infection [1, 2].

Conservative management includes lateral decubitus positioning and selective bronchial intubation. High frequency oscillatory ventilation and neurally adjusted ventilator assist (NAVA) ventilation can also be useful [3–5].

When there is not spontaneous regression or the patient is unstable, surgical treatment should be considered (i.e. lobectomy in located forms) [2]. We evaluated this possibility, but given the extent of the lung injury the only feasible option was to perform a pneumonectomy. Because of the consequences to perform a pneumonectomy and the clinical instability of the patient after a cardiac arrest, we decided to initiate ECMO support and to keep patient ventilated and allow the lung to heal using lung protection strategies during the assistance. There are no actually reports on the use of ECMO in patients with PIE even though its efficacy was described a long time ago [6].

## Conclusions

In summary, we report a 18-day-old male infant with bronchiolitis and PIE associated with mechanical ventilation with high pressures. This disease should be considered in patients with cystic pulmonary lesions and a CT scan should be performed to rule out other congenital or acquired lung diseases. Even though it can be resolved with conservative strategies, sometimes surgical treatment is required, and in our experience, ECMO support could be an option in severe cases.

## Abbreviations

CARE: Guidelines/methodology has been adhered
CPAP: Continuous positive airway pressure
CT: Computed tomographic
ECMO: Extracorporeal Membrane Oxigenation
HFOV: High frequency oscillatory ventilation
PIE: Persistent interstitial emphysema
